# ICTV Virus Taxonomy Profile: Konkoviridae 2026

**DOI:** 10.1099/jgv.0.002290

**Published:** 2026-07-07

**Authors:** Yutaro Neriya, Laura Miozzi, Anna Maria Vaira, Yasuhiro Tomitaka, Takahide Sasaya

**Affiliations:** 1 School of Agriculture, Utsunomiya University, Utsunomiya, 321-8505, Japan; 2 CNR, Institute for Sustainable Plant Protection, Turin, 10135, Italy; 3 Institute for Plant Protection, National Agriculture and Food Research Organization, Tsukuba, 305-8666, Japan; 4 Bio-oriented Technology Research Advancement Institution, National Agriculture and Food Research Organization, Kawasaki, 210-0005, Japan

**Keywords:** International Committee on Taxonomy of Viruses (ICTV) Report, *Konkoviridae*, *Olpivirus*, taxonomy

## Abstract

*Konkoviridae* is a family of negative-sense RNA viruses with genomes of 8.7–10.6 kb that have been associated with plants. Virions are filamentous in the shape of kinked circles. The konkovirus genome consists of three or four segments with three to six ORFs that encode a large protein (L) containing an RNA-directed RNA polymerase domain, a nucleoprotein, a putative cell-to-cell movement protein and additional one to three non-structural proteins of unknown function. This is a summary of the International Committee on Taxonomy of Viruses (ICTV) Report on the family *Konkoviridae*, which is available at ictv.global/report/konkoviridae.

## Virion

**Fig. 1.**
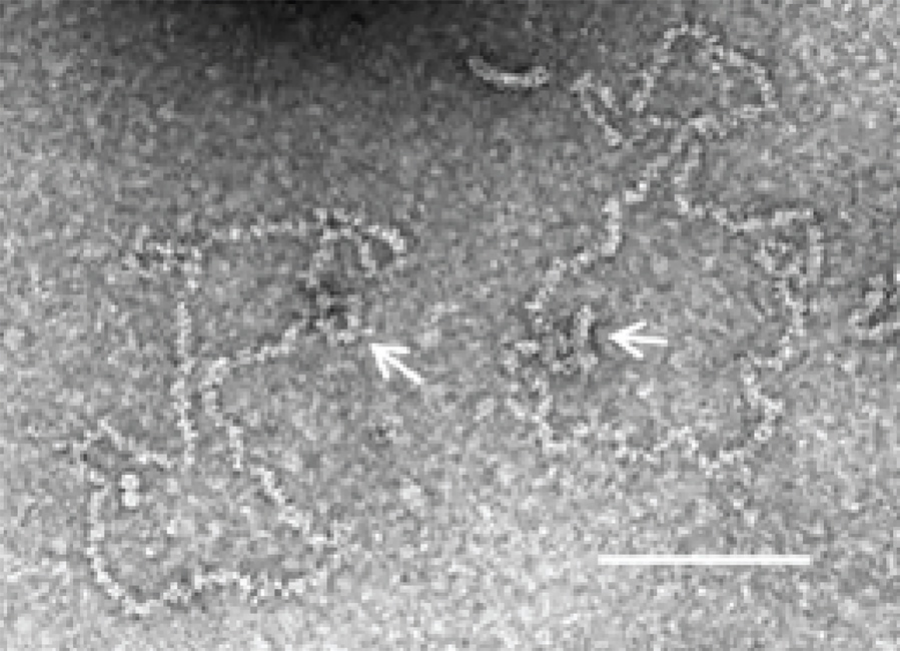
Negative-contrast electron micrograph (uranyl acetate) of tulip streak virus. The bar represents 100 nm. Panhandle forms of the large particles are indicated by arrows (Courtesy of T. Morikawa).

Filamentous particles are spiral-shaped or panhandle-like kinked circles, 7–11 nm in diameter, with lengths (300–1600 nm) proportional to the lengths of the genome RNA segments (Table 1, Fig. 1) [1].

**Table 1.** Characteristics of members of the family *Konkoviridae*

Example	tulip streak virus (RNA1: LC571987; RNA2: LC571988; RNA3: LC805881; RNA4: LC805882), species *Olpivirus tulipae*
Virion	Filamentous virions, 7–11 nm in diameter, 300–1600 nm in length
Genome	Three or four negative-sense or ambisense RNA molecules ranging from 1.1 to 6.4 kb with panhandle termini
Replication	Unknown
Translation	Unknown
Host range	Plants
Taxonomy	Realm *Riboviria*, kingdom *Orthornavirae*, phylum *Negarnaviricota*, class *Bunyaviricetes*, order *Hareavirales*; the genus *Olpivirus* includes >6 species

## Genome

**Fig. 2.**
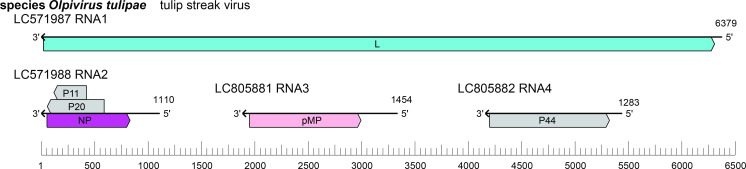
Genome organization of tulip streak virus.

The genome of konkoviruses comprises three or four segments of linear negative-sense or ambisense RNA with a total length of 8.7–10.6 kb (RNA1: 6.3–6.4 kb, RNA2: 1.0–1.3 kb, RNA3: 1.2–1.6 kb and RNA4: about 1.3 kb). The terminal nucleotides of each genome RNA segment are base-paired, possibly forming non-covalently closed ribonucleoprotein complexes. The virus-complementary RNAs 1–4 encode L protein, containing an RNA-directed RNA polymerase domain, nucleocapsid protein, a putative movement protein, a non-structural protein, as well as putative non-structural proteins of unknown function on the virus-sense RNA (Fig. 2) [1,4].

## Replication

Unknown.

## Pathogenicity

Konkoviruses infect plants and cause symptoms such as streaks and necrosis. Tulip streak virus produces streaking symptoms in tulips (*Tulipa gesneriana* L., 1753, Liliaceae) [1] and can be mechanically transmitted using diseased tulip saps to *Nicotiana benthamiana* Domin, 1929, (Solanaceae) and *Chenopodium quinoa* Willd., 1798, (Amaranthaceae). Lactuca big vein-associated phlebovirus causes big-vein symptoms in lettuce (*Lactuca sativa* L., 1753, Asteraceae) [2]. Both these viruses can be transmitted by the soil-inhabiting fungus *Olpidium virulentus* (synonym *Olpidium brassicae*), Olpidiaceae. Additional konkoviruses infect plants in the families Iridaceae, Asparagaceae, Nyctaginaceae and Asteraceae [3,4]. In addition, soil-associated konkovirus has been detected by soil metagenome analysis [3].

## Taxonomy

Current taxonomy: ictv.global/taxonomy. The defining characteristics of konkoviruses are that they form spiral-shaped filamentous virions, have multisegmented, negative-sense or ambisense ssRNA genomes and encode proteins with high sequence identity to proteins of other members of the order *Hareavirales*. Konkoviruses are most closely related to leishbuviruses and phenuiviruses. The family *Konkoviridae* includes at least one genus and seven species of viruses. Additional taxa may be represented among the numerous konkovirus-like sequences that have been detected by metagenomic analysis of non-plant hosts, such as mammals, arthropods and fungi [5].

## Resources

Full ICTV Report on the family *Konkoviridae*: ictv.global/report/konkoviridae.
